# Supplementary material for: Activation of Peroxymonosulfate by Co-Ni-Mo Sulfides/CNT for Organic Pollutant Degradation
Source: Molecules. 2024 Jul 31;29(15):3633. doi: 10.3390/molecules29153633 (PMC11313894; doi:10.3390/molecules29153633)
Supplement: Supplementary file 1 [file molecules-29-03633-s001.zip › molecules-3098196-supplementary.pdf]

Activation of Peroxymonosulfate by Co-Ni-Mo Sulfides/CNT for Organic Pollutant Degradation

Shihao You[1], Jing Di[*1], Tao Zhang[1], , Yufeng Chen[1], Ruiqin Yang[1], Yesong Gao[2], Yin Li[3], Xikun Gai[*1]

1. School of Biological and Chemical Engineering, Zhejiang University of Science and Technology, Hangzhou 310023, China; 212203817017@zust.edu.cn (S.Y.); zhangtaoshd@163.com (T.Z.); 211122050045@zjut.edu.cn (Y.C.); yruiqin@163.com (R.Y.)
2. China Construction Eco-Environmental Group Co., Ltd., Beijing 100037, China; gaosong8208@163.com
3. Ecology and Health Institute, Hangzhou Vocational & Technical College, Hangzhou 310018, China; cherryli1986@126.com

[*]Corresponding authors: dijing@zust.edu.cn (J.D.); gaixikun@zust.edu.cn (X.G.)

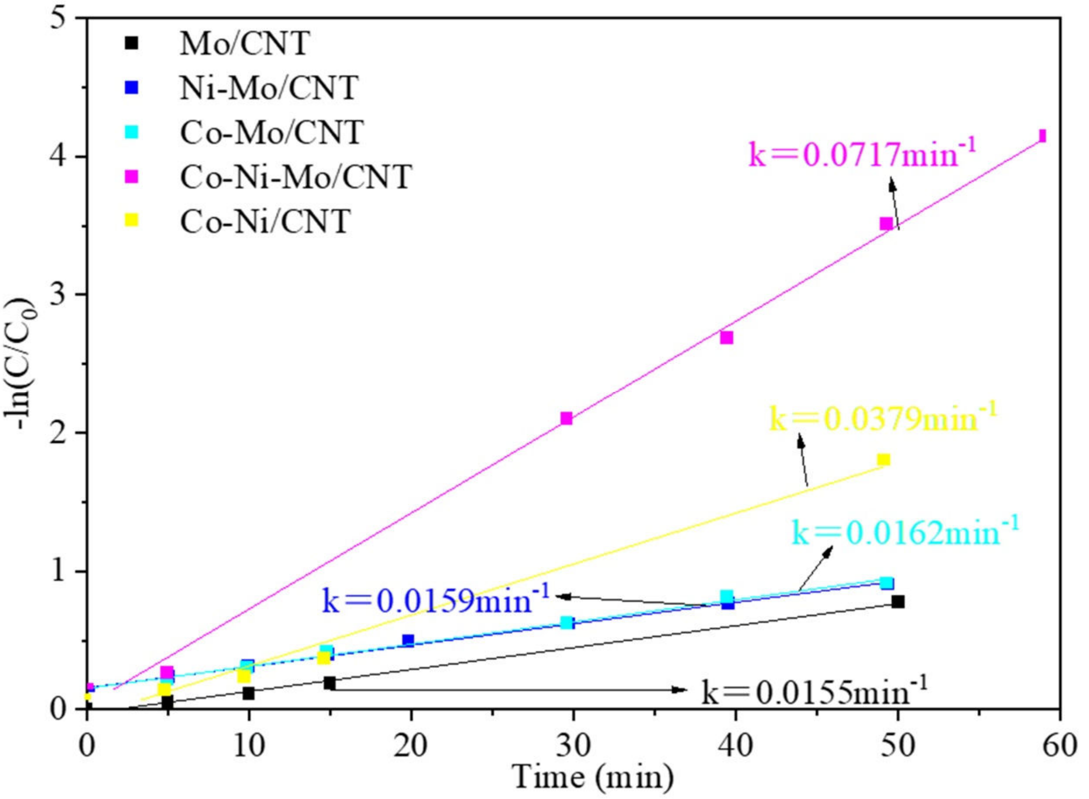

Figure S1. Degradation rate constants of Co-Ni-Mo/CNT, Co-Mo/CNT, Co-Ni/CNT, Ni-Mo/CNT, and Mo/CNT.

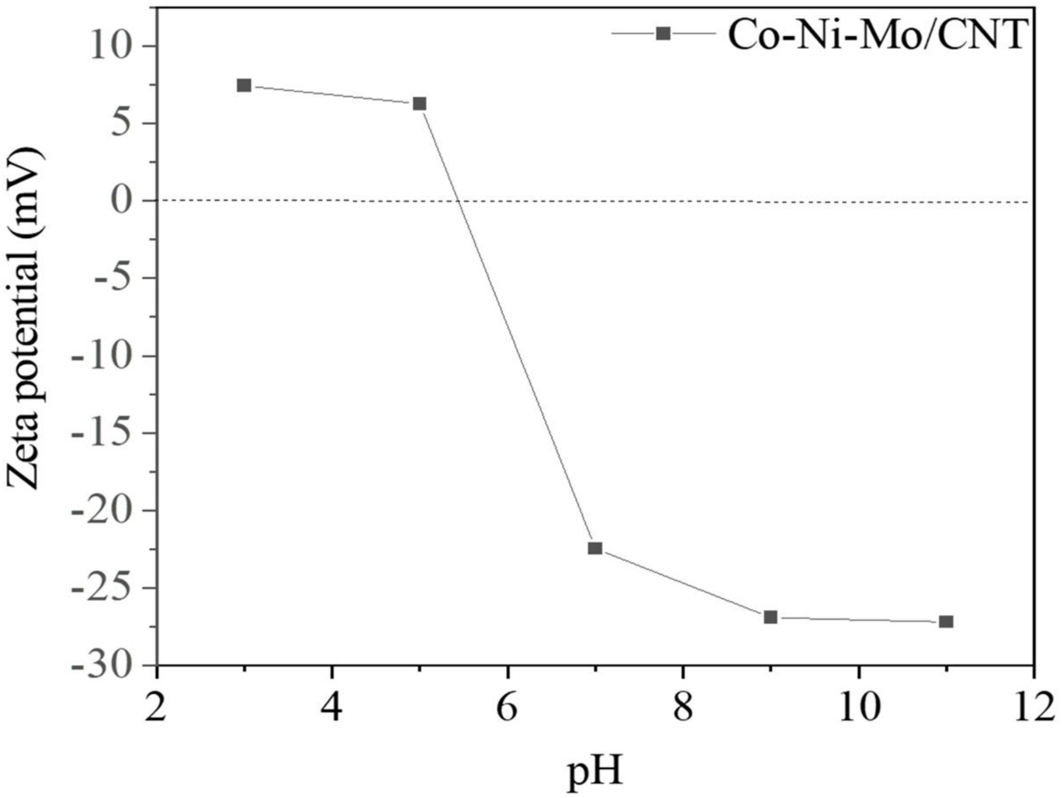

Figure S2. The change of zeta potential of Co-Ni-Mo/CNT with pH.

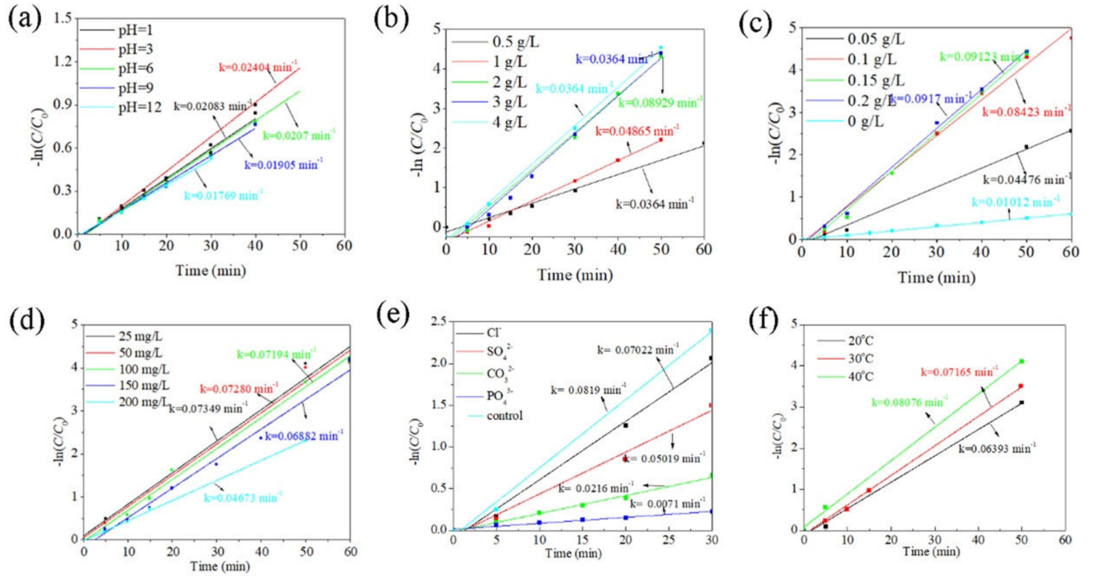

Figure S3. Degradation rate constants on different pH(a), PMS concentration (b), catalyst dosage (c), Rhodamine B concentration(d). different ion species(e), and different temperatures (f).

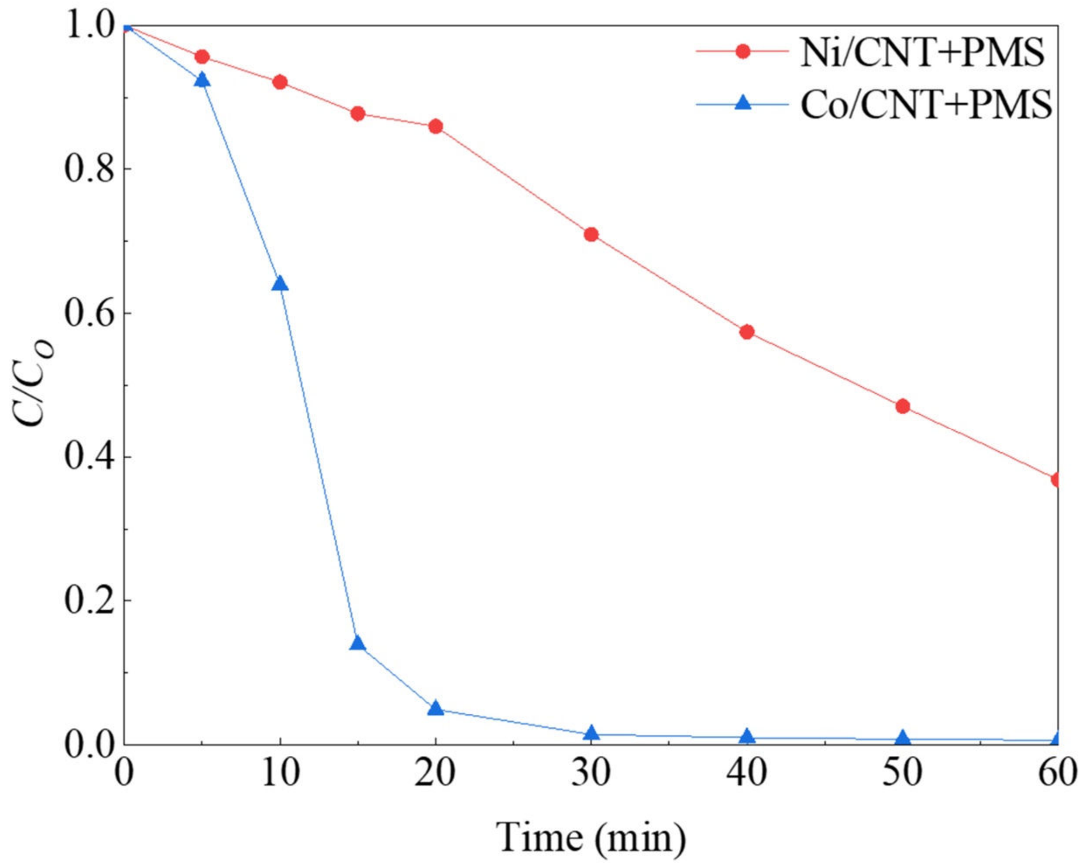

Figure S4. Degradation of Rhodamine B catalyzed by Ni/CNT or Co/CNT.

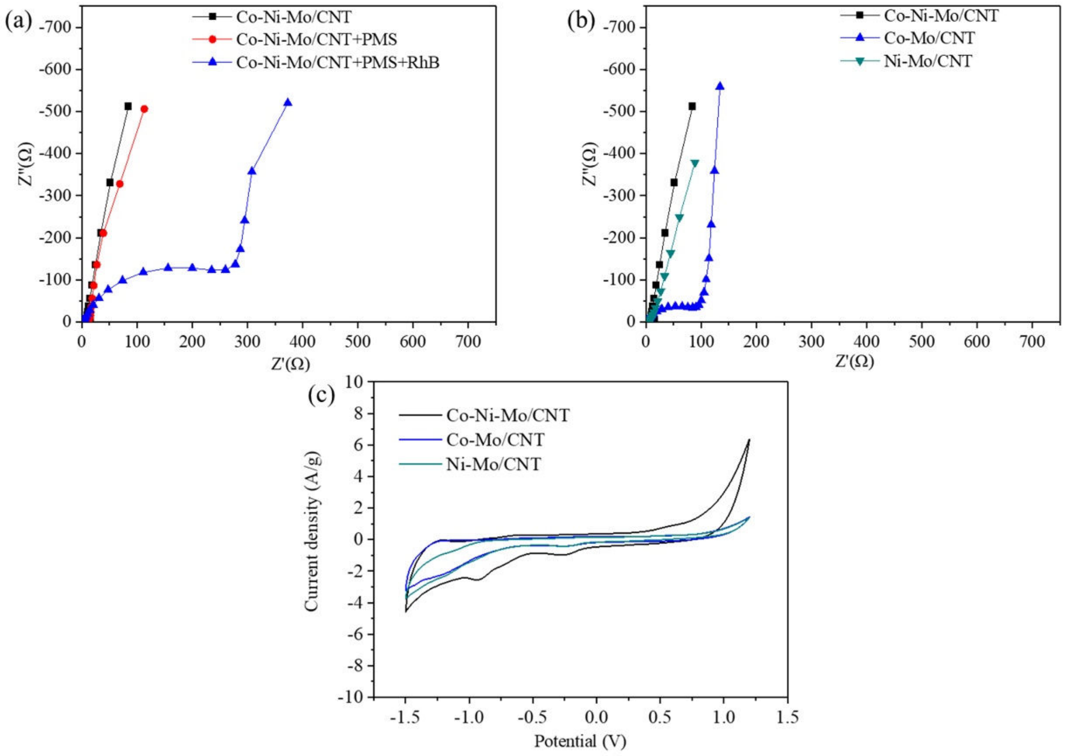

Figure S5. (a) EIS of Co-Ni-Mo/CNT in different systems. (b) EIS and (c) CV curves of Co-Ni-Mo/CNT, Co-Mo/CNT and Ni-Mo/CNT in the presence of PMS.

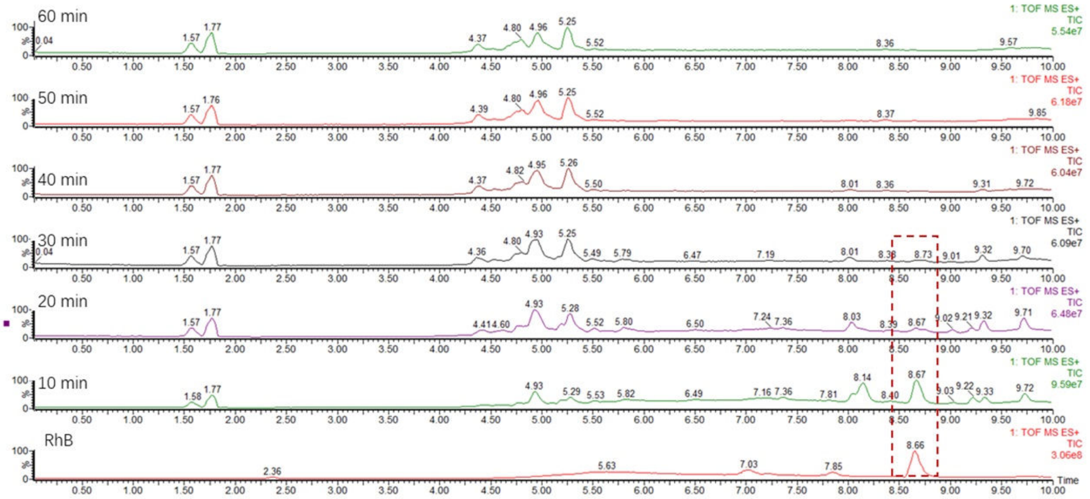

Figure S6. UPLC spectra of Rhodamine B solution at different times in the presence of Co-Ni-Mo/CNT + PMS.

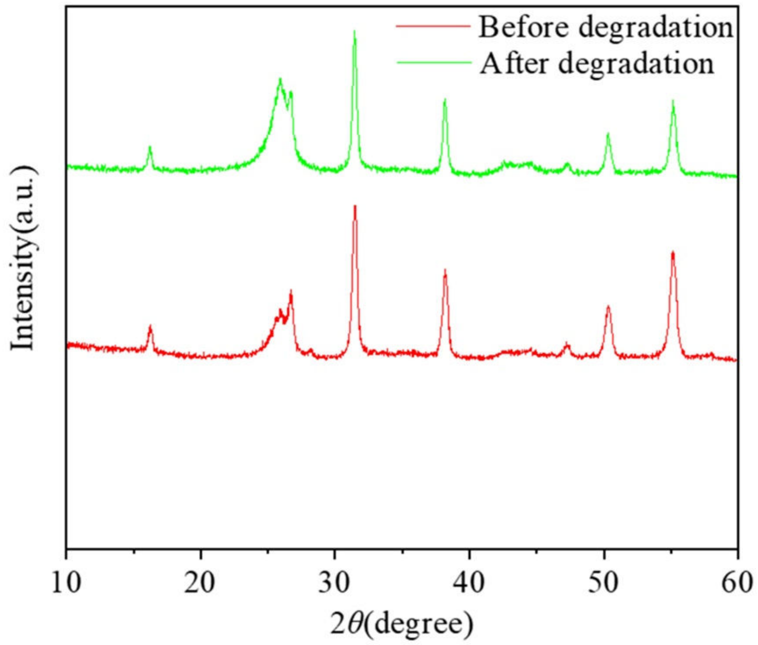

Figure S7. XRD patterns of the recycled catalyst.

Table S1, Intermediates of Rhodamine B degradation in presence of Co-Ni-Mo/CNT + PMS

No.	m/z	Formula	Structure	Intermediates
1	443.2	$C_{28}H_{31}N_2O_3^+$	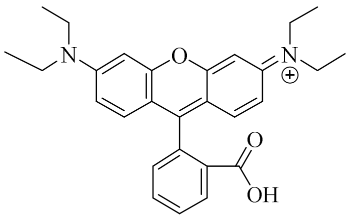	Rhodamine B
2	459.2	$C_{28}H_{31}N_2O_4^+$	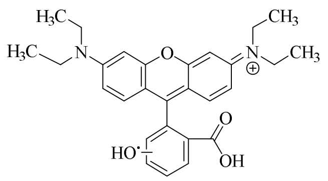	/
3	415.2	$C_{26}H_{27}N_2O_3^+$	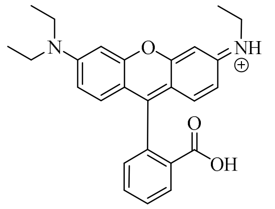	N,N-diethyl-N-ethylrhodamine
4	387.2	$C_{24}H_{23}N_2O_3^+$	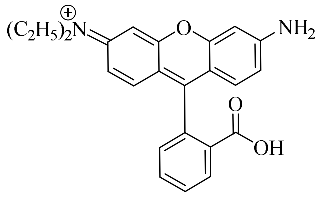	N,N-diethylrhodamine
5	387.2	$C_{24}H_{23}N_2O_3^+$	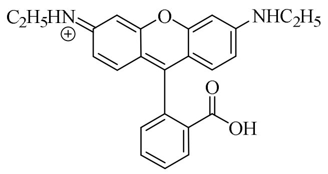	N-ethyl-N-ethylrhodamine
6	290.1	$C_{19}H_{18}N_2O$	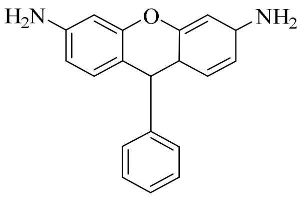	Me-rhodamine
7	258.1	$C_{19}H_{14}O$	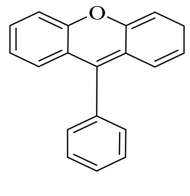	9-phenyl-3H-xanthene
8	246.1	$C_{18}H_{14}O$	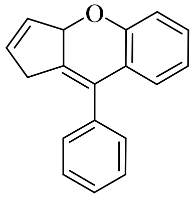	9-phenyl-1,3a-dihydrocyclopenta[b]chromene

9	238.1	$C_{17}H_{18}O$	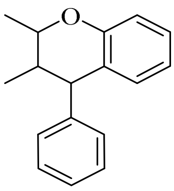	2,3-dimethyl-4-phenyl chromane
10	224.1	$C_{13}H_{20}O_3$	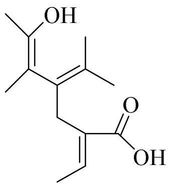	(2E,5Z)-2-ethylidene-6-hydroxy-5-methyl-4-(propan-2-ylidene)hept-5-enoic acid
11	144.1	$C_7H_{12}O_3$	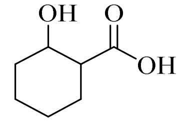	2-hydroxycyclohexane-1-carboxylic acid
12	110.1	$C_7H_{10}O$	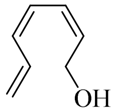	(2Z,4Z)-hepta-2,4,6-trien-1-ol
13	90.0	$C_2H_4O_2$	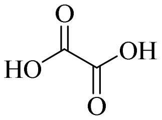	Oxalic acid
14	166.0	$C_8H_6O_4$	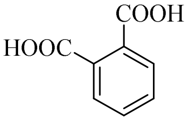	Phthalic acid
15	138.0	$C_7H_6O_3$	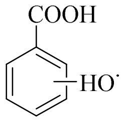	/
16	122.0	$C_7H_6O_2$	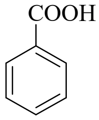	Benzoic acid
